# RAGE-mediated functional DNA methylated modification contributes to cigarette smoke-induced airway inflammation in mice

**DOI:** 10.1042/BSR20210308

**Published:** 2021-06-29

**Authors:** Ping Li, Tao Wang, Mei Chen, Jun Chen, Yongchun Shen, Lei Chen

**Affiliations:** 1 Laboratory of Pulmonary Diseases and Department of Respiratory and Critical Care Medicine, West China Hospital, West China School of Medicine, Sichuan University, Chengdu, Sichuan 610041, P.R. China; 2 Department of Respiratory and Critical Care Medicine, Chengdu Fifth People’s Hospital, Chengdu, Sichuan 611130, P.R. China

**Keywords:** Chronic obstructive pulmonary disease, DNA methylation, Liquid hybridization capture-based bisulfite sequencing, Microarray, Receptor for advanced glycation end products

## Abstract

Our previous study indicated knockout of receptor for advanced glycation end-products (RAGE) significantly attenuated cigarette smoke (CS)-induced airway inflammation in mice. In the present study, we aim to further detect the mediatory effects of RAGE in DNA methylated modification in CS-induced airway inflammation. Lung tissues from the CS-exposed mouse model of airway inflammation were collected for profiling of DNA methylation by liquid hybridization capture-based bisulfite sequencing, which were used for conjoint analysis with our previous data of gene expression by cDNA microarray to identify functional methylated genes, as well as hub genes selected by protein–protein interaction (PPI) network analysis, and functional enrichment analyses were then performed. After RAGE knockout, 90 genes were identified by intersection of the differentially methylated genes and differentially expressed genes. According to the reversed effects of methylation in promoters on gene transcription, 14 genes with functional methylated modification were further identified, among which chemokine (C–X–C motif) ligand 1 (CXCL1), Toll-like receptor 6 (TLR6) and oncostatin M (OSM) with hypomethylation in promoters, were selected as the hub genes by PPI network analysis. Moreover, functional enrichment analyses showed the 14 functional methylated genes, including the 3 hub genes, were mainly enriched in immune-inflammatory responses, especially mitogen-activated protein kinase, tumor necrosis factor, TLRs, interleukin (IL)-6 and IL-17 pathways. The present study suggests that RAGE mediates functional DNA methylated modification in a cluster of 14 targeted genes, particularly hypomethylation in promoters of CXCL1, TLR6 and OSM, which might significantly contribute to CS-induced airway inflammation via a network of signaling pathways.

## Introduction

Chronic obstructive pulmonary disease (COPD) is characterized by persistent respiratory symptoms and airflow limitation that are associated with persistent airway inflammation induced by cigarette smoke (CS), a major causative factor for COPD [1].

Receptor for advanced glycation end-products (RAGE), a membrane protein from the immunoglobulin superfamily, has been implicated in the pathogenesis of COPD [2]. Overexpression of RAGE contributed to airway inflammation in CS-associated COPD [3]. Our previous study further indicated knockout (KO) of RAGE gene significantly attenuated CS-induced airway inflammation in a mouse model [4]. However, the mechanisms regarding the effects of RAGE on airway inflammation in COPD remain not clear. Recently, some evidences implied DNA methylation, an epigenetic regulation, played important roles in RAGE-mediated inflammatory responses in various diseases [5–7], although the role in DNA methylated modification mediated by RAGE in airway inflammation in COPD was not reported.

In consequence, we did the present study, using the established mouse model of CS-induced airway inflammation, to explore the underlying mechanisms of RAGE-mediated DNA methylated modification in airway inflammation in COPD.

## Materials and methods

### Animal model

The animal model of CS-induced airway inflammation has been established in our former study [4]. C57BL/6 mice (7–9 weeks old, 20–22 g weight) were used to generate RAGE KO mice through CRISPR/Cas9 gene targeting technology by Bioray Biotechnology (Shanghai, China). The four experimental groups (*n*=3 mice per group) were included in the present study, as follows: (i) wildtype (WT) group, (ii) CS+WT group, (iii) CS+KO group, (iv) WT+KO group. All mice were specific pathogen-free and kept on a 12-h light/12-h dark cycle, at a room temperature of 22 ± 2°C, with free access to food and water. WT and RAGE KO mice were exposed to mainstream CS or room air for 2 h twice daily, 6 days per week for consecutive 4 weeks. After 4-week CS exposure, the mice were anesthetized intraperitoneally with pentobarbital sodium and killed by femoral artery transection. The animal study was approved by the Panel on Laboratory Animal Care of West China Hospital of Sichuan University and took place at the Experimental Animal Center of West China Hospital of Sichuan University.

### DNA extraction

The total DNA of lung tissues was extracted and purified by DNeasy Blood Tissue Kit (Qiagen), according to the manufacturer’s instructions. Purified DNA was then quantified by NanoDrop2000 Spectrophotometer (Thermo) and agarose gel electrophoresis. Only DNA samples with A_260/280_ ratio between 1.8 and 2.0 were used for further experiments.

### Liquid hybridization capture-based bisulfite sequencing

Genomic DNA (1 μg per sample) was randomly fragmented into approximately 200–300 bp by sonication. After purification, the DNA fragments were repaired in the blunt and phosphorylated ends, which were subsequently 3′ adenylated and then ligated to the methylated adapter using the SureSelect^XT^ Mouse methyl-seq Library Prep Kit (Agilent). After that, the DNA hybridization was performed using the SureSelect™ Methyl-SeQ Hybridization Kit (Agilent), which covered 100 Mb of mouse genomic regions, including CpG islands, Gencode promoters, tissue-specific differential methylated regions (DMRs) and DNase I hypersensitive sites. The hybridized DNA was subsequently bisulfite-treated using the EZ DNA Methylation-Gold™ Kit (Zymo Research) to convert unmethylated cytosine into uracil according to the manufacturer’s instructions. Finally, the treated DNA was amplified by polymerase chain reaction (PCR) and sequenced on Illumina Novaseq PE150.

### DNA methylation data analyses

The Fastp software (version 1.2.1) was used to process methylated raw data and remove the low quality reads, including (i) contaminated sequences; (ii) the Q-value of 3′ end is less than 20; (iii) reads with less than 15 bp; (iv) reads with 40% base Q-value less than 15; (v) reads containing N bases greater than 5 (Q = −10*log10(*P*), *P* is the probability of error) [8]. The clean reads were aligned to the reference genome using Bismark software (version 0.19.0) with bowtie2 (version 2.3.4.2) to attain the methylated type, status and proportion [9]. DMRs were subsequently analyzed using the R package methylKit (version 1.6.1) [10] and eDMR [11], with an adjusted *P*-value <0.05 and absolute differential methylation levels (absolute meth. diff > 5%). Finally, related differential methylated genes (DMGs) were located and annotated in the DMRs by the ChIPseeker software.

### cDNA microarray

The data of gene expression by cDNA microarray have been obtained in our former study [4]. In the present study, differentially expressed genes (DEGs) were identified by fold-change, and only genes that at least 1.2-fold up-regulated or down-regulated were analyzed.

### Candidate genes selection

**Figure 1:**
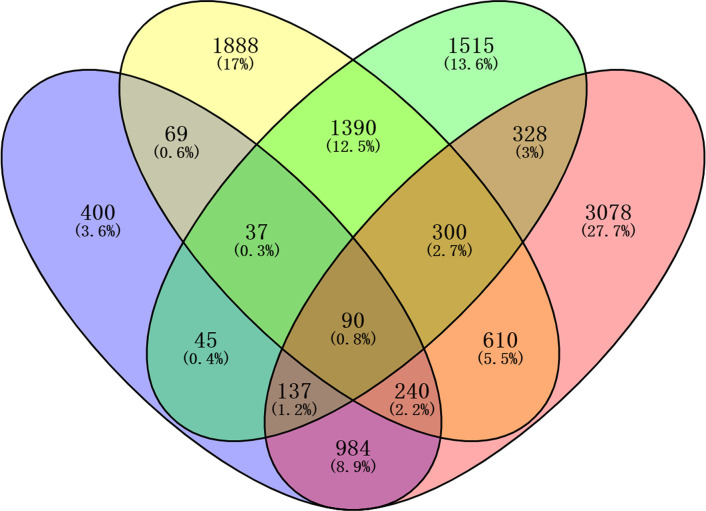
Venn diagram of the intersection model The colorful oval shapes represent CS-associated DEGs (yellow), CS-associated DMGs (blue), RAGE-associated DEGs (green) and RAGE-associated DMGs (purple).

To identify the candidate genes with methylated modification mediated by RAGE, a novel intersection model was performed (Figure 1). Briefly, CS-associated (CS+WT vs WT) and RAGE-associated (CS+KO vs CS+WT plus WT+KO vs WT) DMGs intersected to get the overlapped DMGs, and so did CS-associated and RAGE-associated DEGs (overlapped DEGs). Then, the overlapped DMGs and DEGs intersected again to select the candidate genes.

### Functional methylated genes and hub genes selection

The candidate genes with functional methylated modification, also called functional methylated genes, were identified according to the reversed effects of methylation in promoters on gene transcription. To further identify the hub genes that were most correlated with other genes, protein–protein interaction (PPI) network analysis among the functional methylated genes was performed using the online database STRING [12] and displayed using Cytoscape [13].

### Functional enrichment analyses

Functional enrichment analyses on the functional methylated genes, as well as the hub genes, using Gene Ontology (GO) and Kyoto Encyclopedia of Genes and Genomes (KEGG) databases were performed with the clusterProfiler package using *P*-value <0.05 was set as the threshold.

## Results

### Data production

Based on the liquid hybridization capture-based bisulfite sequencing (LHC-BS) method, 15 Gbp raw sequence data were generated on average for each sample. More than 79% were mapped to at less one genomic position, covering ∼77% of the target regions, with an average of 57× sequencing depth per CpG and 12.5% duplication data, and the duplicated sequence reads were filtered for the subsequent analyses.

### RAGE-mediated functional DNA methylated modification in targeted genes in CS-induced airway inflammation

**Figure 2:**
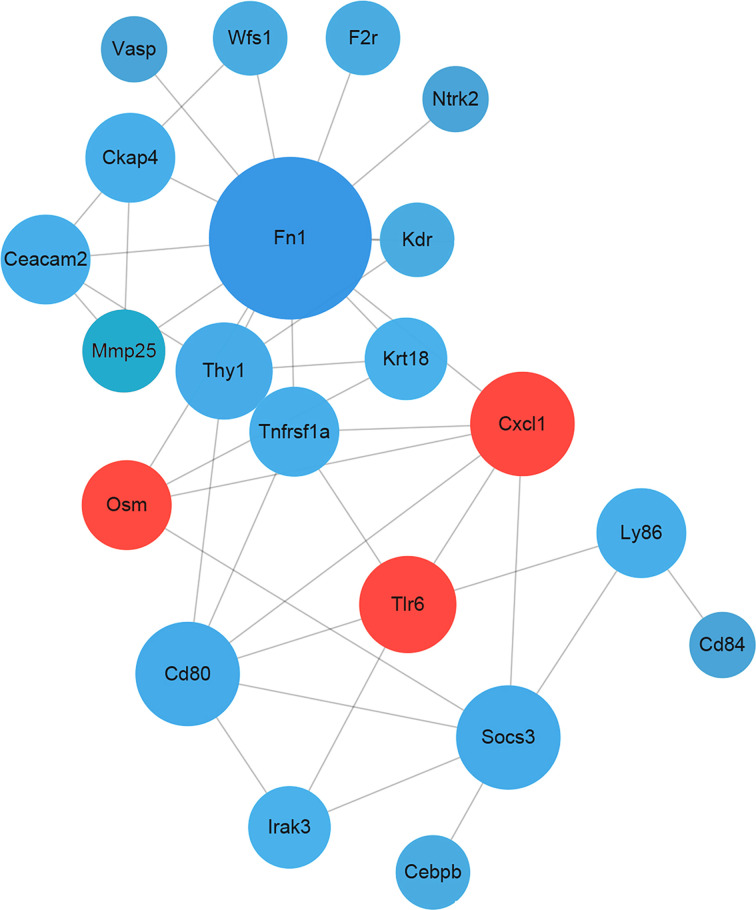
PPI network analysis of the candidate genes Edges indicate correlations between two genes. The bigger circles indicate more related genes and red circles indicate the hub genes.

After RAGE KO, as reported in our previous study, CS-induced airway inflammation was significantly improved [4]. Meanwhile, 90 overlapping candidate genes were identified via the intersection of DMGs with DEGs (Supplementary Table S1). It is well-known that DNA methylation occurs almost in CpG islands that are primarily located in the regions of promoter and are negatively correlated with gene expression [14]. As a result, 14 genes from the 90 candidate genes, with functional methylated modification in promoters were identified (Table 1), among which, three genes, including chemokine (C–X–C motif) ligand 1 (CXCL1), Toll-like receptor 6 (TLR6) and oncostatin M (OSM) with hypomethylation in promoters were finally selected as the hub genes by PPI network analysis (Figure 2). Furthermore, GO and KEGG analyses indicated the 14 functional methylated genes, including the 3 hub genes, were significantly enriched in immune-inflammatory responses, especially mitogen-activated protein kinase (MAPK), tumor necrosis factor (TNF), TLRs, interleukin (IL)-6 and IL-17 signaling pathways (Supplementary Tables S2 and S3).

**Table 1:** The 14 functional methylated genes

Gene	Description	Genomic localization	Methylation change^*^	Fold change
*Alox5ap*	Aachidonate 5-lipoxygenase activating protein	Promoter Distal intergenic 3′ UTR	Hyper (15.6131412) Hypo (−12.032621795) Hypo (−13.27280036)	−1.2
*Arhgap22*	Rho GTPase activating protein 22	Promoter Promoter	Hyper (10.29366302) Hypo (−6.476953839)	−1.3
*Cxcl1*	Chemokine (C–X–C motif) ligand 1	Promoter	Hyper (10.2010746)	−1.2
*Dmc1*	DNA meiotic recombinase 1	Promoter	Hypo (9.450360901)	1.2
*Inf2*	Iverted formin, FH2 and WH2 domain containing	Promoter Distal intergenic	Hyper (13.93768033) Hyper (13.60386215)	−1.2
*Mir1981*	MicroRNA 1981	Promoter	Hypo (−6.417671883)	1.2
*Mmp25*	Matrix metallopeptidase 25	Promoter Promoter	Hyper (15.40303433) Hypo (−13.06629416)	−1.4
*Osm*	Oncostatin M	Promoter	Hyper (15.05770235)	−1.4
*Psmb9*	Proteasome 20S subunit β 9	Promoter	Hyper (10.74327874)	−1.3
*Rnaseh2b*	Ribonuclease H2, subunit B	Promoter	Hyper (11.78927527)	−1.3
*Slc16a6*	Solute carrier family 16 member 6	Promoter Intron	Hyper (8.126762879) Hyper (8.360891276)	−1.2
*Srgn*	Srglycin	Promoter	Hyper (9.932392771)	−1.3
*Tlr6*	Toll-like receptor 6	Promoter	Hyper (17.51844104)	−1.4
*Tmod2*	Tropomodulin 2	Promoter	Hypo (−7.597567585)	1.3

* Methylation changes (%) in gene expression by CS+KO vs CS+WT. Abbreviations: Hyper, hypermethylation; Hypo, hypomethylated; Inc, increased variance.

## Discussion

DNA methylation is closely associated with COPD susceptibility, exacerbation and lung function decline [15,16]. In this process, RAGE may play a regulatory role in DNA methylated modification in COPD, whereas the mechanisms remain unexplained. Consequently, in the present study, the 90 DMGs, regarding RAGE-mediated airway inflammation induced by CS exposure, were initially selected using a novel intersection model, and the functional DNA methylated modification in 14 targeted genes were subsequently identified, especially hypomethylation in CXCL1, TLR6 and OSM promoters, which might significantly contribute to RAGE-mediated airway inflammation in COPD via a network of signaling pathways, such as MAPK, TNF, TLRs, ILs, etc.

According to the GO and KEGG analyses, the majority of the candidate genes, especially the 14 functional methylated genes widely participated in CS-associated inflammatory-immune responses. In particular, the three hub genes were documented to play important roles in the inflammatory process in COPD. CXCL1 is a member of chemokine subfamily of CXC [17] and the increased level of CXCL1 was detected in the lungs of COPD [18]. CXCL1 served as a chemoattractant for neutrophils migrating from circulation to respiratory tracts, which contributed to neutrophilic inflammation of COPD [19]. TLR6 belongs to the Toll-like receptor family [20], which initiates innate immune responses in airway epithelial cells and triggers inflammatory responses [21]. Furthermore, TLRs activation could increase CXCL1 production in human pulmonary macrophages [22]. OSM, a member of IL-6 subfamily [23], participates in a variety of inflammatory diseases with a high level [24–26]. In COPD, the inflammatory mechanisms for OSM may be stimulating IL-6 production and IL-6 related inflammation, which is positively correlated with pulmonary function decline [23,27]. Noticeably, as suggested in our study, CXCL1, TLR6 and OSM have been documented to be targeted genes of DNA methylated modification in other diseases, such as schizophrenia [28], type 1 diabetes [29] and Richter syndrome [30].

However, some limitations in the present study should be considered. First, the sample size of mice in each group was relatively small, although the minimum requirement for biological repeat was reached. Second, the novel intersection model might be theoretically imperfect. Third, validation was thus needed in future studies.

In summary, the present study performed intersections of DMGs with DEGs in a CS-exposed mouse model and indicated RAGE could mediate functional methylated modification in multiple targeted genes, especially CXCL1, TLR6 and OSM, which might significantly contribute to airway inflammation in COPD.

## Supplementary Material

Supplementary Tables S1-S3

## Data Availability

The data presented in this manuscript are available from the corresponding author (Lei Chen) on reasonable request.

## Abbreviations

COPD: chronic obstructive pulmonary disease
CS: cigarette smoke
CXCL1: chemokine (C–X–C motif) ligand 1
DEG: differentially expressed gene
DMG: differential methylated gene
DMR: differential methylated region
GO: Gene Ontology
IL: interleukin
KEGG: Kyoto Encyclopedia of Genes and Genomes
KO: knockout
MAPK: mitogen-activated protein kinase
OSM: oncostatin M
PPI: protein–protein interaction
RAGE: receptor for advanced glycation end-product
TLR: Toll-like receptor
TNF: tumor necrosis factor
WT: wildtype
