# Supplementary material for: RAGE-mediated functional DNA methylated modification contributes to cigarette smoke-induced airway inflammation in mice
Source: Biosci Rep. 2021 Jun 29;41(7):BSR20210308. doi: 10.1042/BSR20210308 (PMC8243334; doi:10.1042/BSR20210308)
Supplement: Supplementary Tables S1-S3 [file BSR-2021-0308_supp.pdf]

Table S1. The 90 candidate genes selected according to the methylation and gene expression

Gene	Description	Genomic localization	Methylation change*	Fold Change
1600014C10Rik	RIKEN cDNA 1600014C10 gene	Promoter/Intron	Hypo/Hyper	-1.3
2610035D17Rik	RIKEN cDNA 2610035D17 gene	Intron	Inc	1.3
2610528J11Rik	RIKEN cDNA 2610528J11 gene	Promoter/Distal Intergenic	Hypo/Hypo	-1.3
Agpat4	1-acylglycerol-3-phosphate O-acyltransferase 4	Intron	Inc	-1.3
Alox5ap	arachidonate 5-lipoxygenase activating protein	Promoter/Distal Intergenic/3' UTR	Hyper/Hypo/Hypo	-1.2
Arhgap22	Rho GTPase activating protein 22	Promoter	Inc	-1.3
Atoh8	atonal bHLH transcription factor 8	Intron /Distal Intergenic	Hypo/Hypo	-1.7
Best2	bestrophin 2	3' UTR	Hypo	1.2
Blm	Bloom syndrome, RecQ like helicase	Exon/3' UTR	Inc/Hypo	-1.2
Btg3	B cell translocation gene 3	Intron/Distal Intergenic	Hyper/Hyper	-1.6
Cd80	CD80 antigen	Intron/Exon	Hypo/Hypo	-1.3
Cd84	CD84 antigen	Promoter /Intron/Distal Intergenic/3' UTR	Hyper/Hyper/Hyper/Hypo	1.3
Cdc6	cell division cycle 6	3' UTR	Hypo	-1.3
Ceacam2	carcinoembryonic antigen-related cell adhesion molecule 2	Intron	Hyper	1.3
Cebpb	CCAAT/enhancer binding protein (C/EBP), beta	Promoter/Intron	Hypo/Hyper	-1.3
Ckap4	cytoskeleton-associated protein 4	Distal Intergenic	Hyper	-1.4
Cxcl1	chemokine (C-X-C motif) ligand 1	Promoter	Hyper	-1.2
Dmc1	DNA meiotic recombinase 1	Promoter	Hypo	1.2
Dusp6	dual specificity phosphatase 6	Exon/Distal Intergenic	Hyper/Hyper	-1.7
Ear1	eosinophil-associated, ribonuclease A family, member 1	Distal Intergenic	Hyper	1.7
Emilin2	elastin microfibril interfacer 2	Exon/Intron/Downstream	Hyper/Inc/Hyper	-1.5
F2r	coagulation factor II (thrombin) receptor	Exon/Distal Intergenic/Downstream	Hyper/Hyper/Hyper	-1.3

Fn1	fibronectin 1	Exon/Distal Intergenic	Hyper/Hypo	-1.7
Galnt15	polypeptide N-acetylgalactosaminyltransferase 15	Promoter	Hypo	-1.3
Galnt2	polypeptide N-acetylgalactosaminyltransferase 2	Intron	Hyer	-1.3
Galnt9	polypeptide N-acetylgalactosaminyltransferase 9	Intron	Inc	-1.3
Gclc	glutamate-cysteine ligase, catalytic subunit	Distal Intergenic	Hypo	1.6
Gdf10	growth differentiation factor 10	Intron	Hyper	-1.3
Gm3696	predicted gene 3696	Intron	Hyper	1.6
Gm5148	predicted gene 5148	Distal Intergenic	Inc	-1.2
Gm6548	eukaryotic translation elongation factor 1 alpha 1 pseudogene	Promoter	Hyper	1.3
Gm8300	predicted gene 8300	Distal Intergenic	Hyper	1.6
Gp6	glycoprotein 6 (platelet)	Distal Intergenic	Hyper	-1.2
Gsg1l	GSG1-like	Promoter	Hyper	1.2
Ifitm2	interferon induced transmembrane protein 2	Promoter	Hypo	-1.5
Inf2	inverted formin, FH2 and WH2 domain containing	Promoter/Distal Intergenic	Hyper/Hyper	-1.2
Irak3	interleukin-1 receptor-associated kinase 3	Intron	Inc	-1.3
Kcnj8	potassium inwardly-rectifying channel, subfamily J, member 8	Distal Intergenic	Inc	-1.3
Kdr	kinase insert domain protein receptor	Exon/Distal Intergenic	Hyper/Hypo	1.4
Krt18	keratin 18	Promoter/Distal Intergenic	Hypo/Hypo	-1.5
Ldb2	LIM domain binding 2	Intron	Hyper	-1.2
Lgi2	leucine-rich repeat LGI family, member 2	Distal Intergenic	Hypo	-1.2
Lmnb1	lamin B1	Distal Intergenic	Hyper	-1.2
Ly86	lymphocyte antigen 86	Exon/Distal Intergenic	Hyper/Hypo	-1.2
Map3k7cl	Map3k7 C-terminal like	Distal Intergenic	Hyper	-1.3
Mctp1	multiple C2 domains, transmembrane 1	Intron	Hyper	-1.5

Mir1981	microRNA 1981	Promoter	Hypo	1.2
Mmp25	matrix metallopeptidase 25	Promoter	Inc	-1.4
Mthfd1l	methylenetetrahydrofolate dehydrogenase (NADP+ Dependent) 1 Like	Exon	Hypo	-1.4
Myo18b	myosin XVIIIb	Intron/Exon	Hypo/Hypo	1.2
Nav2	neuron navigator 2	Intron	Inc	1.2
Ncf1	neutrophil cytosolic factor 1	Intron/3' UTR	Hypo/Hypo	-1.3
Ntrk2	neurotrophic tyrosine kinase, receptor, type 2	Intron	Hypo	-1.6
Nupl2	nucleoporin like 2	Intron	Hyper	1.3
Osm	oncostatin M	Promoter	Hyper	-1.4
Padi4	peptidyl arginine deiminase, type IV	Exon/Downstream	Hyper/Hypo	-1.6
Pgam2	phosphoglycerate mutase 2	Promoter	Hyper	1.3
Pgm1	phosphoglucomutase 1	Exon/Distal Intergenic	Hyper/Hyper	-1.4
Pln	phospholamban	Distal Intergenic	Hyper	1.5
Ppargc1a	peroxisome proliferative activated receptor, gamma, coactivator 1 alpha	Distal Intergenic	Hypo	-1.3
Prkar2b	protein kinase, cAMP dependent regulatory, type II beta	Intron/Exon/Distal Intergenic	Hyper/Hyper/Hyper	1.4
Psmb9	proteasome 20S subunit beta 9	Promoter	Hyper	-1.3
Pttg1	pituitary tumor-transforming gene 1	Promoter	Hyper	1.3
Rcsd1	RCSD domain containing 1	Distal Intergenic	Hypo	-1.2
Rerg	RAS-like, estrogen-regulated, growth-inhibitor	Promoter/Intron	Hypo/Hyper	-1.2
Rfc5	replication factor C (activator 1) 5	Distal Intergenic	Hyper	-1.3
Rnaseh2b	ribonuclease H2, subunit B	Promoter	Hyper	-1.3
Rps9	ribosomal protein S9	Promoter /Distal Intergenic	Hyper/Hypo	1.3
Rrm2	ribonucleotide reductase M2	Exon/Distal Intergenic	Hyper/Hyper	-1.3
Slc16a6	Solute Carrier Family 16 Member 6	Promoter/Intron	Hyper/Hyper	-1.2

Slc1a5	solute carrier family 1 member 5	Exon/Distal Intergenic	Inc/Hyper	1.3
Slc7a5	solute carrier family 7 member 5	Distal Intergenic	Hyper	-1.5
Slc7a8	solute carrier family 7 member 8	Intron/Distal Intergenic	Hyper/Hyper	-1.3
Socs3	suppressor of cytokine signaling 3	Distal Intergenic	Hyper	-1.6
Spata18	spermatogenesis associated 18	Distal Intergenic	Inc	1.5
Srgn	serglycin	Promoter	Hyper	-1.3
Srp9	signal recognition particle 9	Intron/Distal Intergenic	Hyper/Inc	-1.2
St3gal1	ST3 beta-galactoside alpha-2,3-sialyltransferase 1	Intron /Distal Intergenic	Hyper/Hyper	-1.2
Tbc1d30	TBC1 domain family, member 30	Promoter/Exon/3' UTR	Hyper/Hypo/Hyper	1.2
Thy1	thymus cell antigen 1, theta	Promoter	Hypo	-1.3
Timm22	translocase of inner mitochondrial membrane 22	Intron/Exon	Hypo/Hypo	-1.4
Tlr6	toll-like receptor 6	Promoter	Hyper	-1.4
Tmem45b	transmembrane protein 45b	Distal Intergenic	Inc	1.5
Tmod2	tropomodulin 2	Promoter	Hypo	1.3
Tnfrsf1a	tumor necrosis factor receptor superfamily, member 1a	3' UTR	Hypo	-1.2
Vasp	vasodilator-stimulated phosphoprotein	Downstream	Hyper	-1.3
Vmn2r33	vomeronasal 2, receptor 33	Distal Intergenic	Hyper	1.6
Wfs1	wolframin ER transmembrane glycoprotein	Intron	Hyper	-1.3
Zbp1	Z-DNA binding protein 1	Intron	Hypo	-1.3
Zbtb20	zinc finger and BTB domain containing 20	Intron	Hypo	1.3

*Methylation changes with fold changes in gene expression by CS+KO vs CS+WT. Hyper, hypermethylation; Hypo, hypomethylated; Inc, increased variance.

Table S2. GO enrichment analysis (the functional methylated genes marked red)

Cellular component/Biologic process/Molecular function	Genes	Count	P value
regulation of MAPK cascade	Osm, Tlr6, Irak3, F2r, Fn1, Gdf10, Ncf1, Lmnb1, Map3k7cl, Kdr, Dusp6, Ntrk2	12	1.40729E-05
regulation of interleukin-6 production	Tlr6, F2r, Zbtb20, Cebpb, Irak3	5	2.34084E-05
MAPK cascade	Osm, Tlr6, Irak3, F2r, Map3k7cl, Fn1, Ncf1, Kdr, Gdf10, Dusp6, Ntrk2, Lmnb1	12	3.43092E-05
inflammatory response	Cxcl1, Alox5ap, Mmp25, Tlr6, F2r, Tnfrsf1a, Ly86, Fn1, Ncf1, Socs3	10	9.48037E-05
defense response	Tlr6, Mmp25, Cxcl1, Alox5ap, Padi4, Kcnj8, F2r, Zbp1, Socs3, Tnfrsf1a, Ly86, Fn1, Ncf1, Cebpb, rak3, Ifitm2	16	0.000251799
positive regulation of MAPK cascade	Osm, Tlr6, F2r, Lmnb1, Map3k7cl, Kdr, Ncf1, Ntrk2	8	0.000312195
response to cytokine	Cxcl1, Osm, Ppargc1a, Ifitm2, Zbp1, Irak3, Cebpb, Krt18, Tnfrsf1a, Gclc, Socs3	11	0.000511441
negative regulation of cytokine production	Tlr6, Srgn, Irak3, Cd84, Fn1	5	0.000563016
cytokine-mediated signaling pathway	Cxcl1, Osm, Irak3, Zbp1, Socs3, Tnfrsf1a, Krt18	7	0.000723893
regulation of cytokine production	Srgn, Tlr6, F2r, Irak3, Cd84, Fn1, Cebpb, Zbtb20	8	0.001398345
regulation of cytokine secretion	Srgn, Tlr6, Fn1, F2r	4	0.001544646
cellular response to cytokine stimulus	Cxcl1, Osm, Socs3, Krt18, Tnfrsf1a, Cebpb, Ppargc1a, Zbp1, Irak3	9	0.002115125
cytokine secretion	Tlr6, Srgn, Fn1, F2r	4	0.002629006
cytokine production	Tlr6, Srgn, Irak3, Cd84, F2r, Cebpb, Fn1,	8	0.002833485

	Zbtb20		
regulation of MAP kinase activity	Tlr6, F2r, Irak3, Map3k7cl, Dusp6	5	0.002862216
immune system process	Psmb9, Tlr6, Cxcl1, Osm, Galnt2, Kcnj8, Zbp1, Tnfrsf1a, Ncf1, Padi4, Cd80, Cd84, Thy1, Blm, Irak3, Ifitm2, Ly86, Cebpb	18	0.008472928
negative regulation of immune system process	Tlr6, Cd84, Irak, Thy1, Cebpb	5	0.015585279
positive regulation of immune system process	Tlr6, Cxcl1, Cd80, Blm, Cd84, Irak3, Zbp1, Thy1	8	0.024008534
immune response	Osm, Tlr6, Cxcl1, Tnfrsf1a, Ly86, Ifitm2, Irak3, Padi4, Thy1, Zbp1,Cd84	11	0.031630554

Table S3 KEGG enrichment analysis

Pathways	Count	Genes	P value
TNF signaling pathway	4	Cxcl1, Socs3, Tnfrsf1a, Cebpb	0.00164
DNA replication	2	Rnaseh2b, Rfc5	0.00333
IL-17 signaling pathway	2	Cxcl1, Cebpb	0.04311
Toll-like receptor signaling pathway	2	Tlr6, Cd80	0.05491

(the functional methylated genes marked red)
